# The mechanism of dysbiosis in alcoholic liver disease leading to liver cancer

**DOI:** 10.20517/2394-5079.2019.29

**Published:** 2020-02-20

**Authors:** Nahum Méndez-Sánchez, Alejandro Valencia-Rodriguez, Alfonso Vera-Barajas, Ludovico Abenavoli, Emidio Scarpellini, Guadalupe Ponciano-Rodriguez, David Q.-H. Wang

**Affiliations:** 1 Liver Research Unit, Medica Sur Clinic & Foundation, Mexico City 14050, Mexico; 2 Faculty of Medicine, National Autonomous University of Mexico, Mexico City 04510, Mexico; 3 Department of Health Sciences, University “Magna Graecia” Viale Europa, Catanzaro 88100, Italy; 4 Clinical Nutrition Unit, and Internal Medicine Unit, “Madonna del Soccorso” General Hospital, Via Luciano Manara 7, San Benedetto del Tronto (AP) 63074, Italy; 5 Public Health Department, Faculty of Medicine, National Autonomous University of Mexico, Mexico City 04510, Mexico; 6 Department of Medicine and Genetics, Division of Gastroenterology and Liver Diseases, Marion Bessin Liver Research Center, Einstein-Mount Sinai Diabetes Research Center, Albert Einstein College of Medicine, Bronx, NY 10461, USA

**Keywords:** Alcoholic liver disease, gut microbiota, dysbiosis, hepatocellular carcinoma

## Abstract

Currently, alcoholic liver disease (ALD) is one of the most prevalent chronic liver diseases worldwide, representing one of the main etiologies of cirrhosis and hepatocellular carcinoma (HCC). Although we do not know the exact mechanisms by which only a selected group of patients with ALD progress to the final stage of HCC, the role of the gut microbiota within the progression to HCC has been intensively studied in recent years. To date, we know that alcohol-induced gut dysbiosis is an important feature of ALD with important repercussions on the severity of this disease. In essence, an increased metabolism of ethanol in the gut induced by an excessive alcohol consumption promotes gut dysfunction and bacterial overgrowth, setting a leaky gut. This causes the translocation of bacteria, endotoxins, and ethanol metabolites across the enterohepatic circulation reaching the liver, where the recognition of the pathogen-associated molecular patterns via specific Toll-like receptors of liver cells will induce the activation of the nuclear factor kappa-B pathway, which releases pro-inflammatory cytokines and chemokines. In addition, the mitogenic activity of hepatocytes will be promoted and cellular apoptosis will be inhibited, resulting in the development of HCC. In this context, it is not surprising that microbiota-regulating drugs have proven effectiveness in prolonging the overall survival of patients with HCC, making attractive the implementation of these drugs as co-adjuvant for HCC treatment.

## INTRODUCTION

The metabolic effects of alcohol in humans has been a topic of great interest for many years due to the important relationship between excessive alcohol consumption and disease even reaching to cancer development. In this context, The Global Burden of Disease Study 2015 reported the primary liver cancer incidence, mortality, and disability-adjusted life-years of 195 countries from 1990 to 2015. Surprisingly, the cases of incident liver cancer increased by 75% between 1990 and 2015. Alcohol-induced liver cancer globally accounted for 245,000 (30%) deaths with important variations between countries and sex. Only in 2015, alcohol caused 204,000 [95% uncertainty interval (UI), 177,000-240,000] liver cancer cases in men and 45,000 (95% UI, 38,000-54,000) cases among women. Eastern Europe was the geographical region which contributed with the most alcohol-induced liver cancer cases in the world, accounting for 53% of them^[1]^. According to WHO statistics, alcohol is involved in more than 200 different diseases^[2]^. Among them, gastrointestinal (GI) disorders (mainly cirrhosis) represent the third cause in mortality secondary to excessive alcohol consumption^[3]^. Interestingly, the metabolism of alcohol goes beyond the liver; in recent years, the role of the gut-liver axis in the development and aggravation of alcoholic liver disease (ALD) has emerged as an important element to consider^[4,5]^. The gut microbiota and a selected group of catalytic enzymes of the GI tract are key elements in ethanol metabolism and its passage to systemic circulation. Furthermore, evidence has shown carcinogenic effects of different alcohol and gut metabolites in ALD patients, bringing new perspectives in the development of hepatocellular carcinoma (HCC) in this group of subjects. For this reason, this review discusses in a systematic way the role of alcohol-induced dysbiosis in the development of ALD and its progression to HCC, starting with the different metabolic pathways of ethanol within the human body and its deregulation in chronic alcohol consumption. Then, the mechanisms of alcohol-induced dysbiosis with the consequent liver injury and hepatocarcinogenesis are addressed and finally the future perspectives of microbiota-regulating drugs as adjuvants for HCC treatment are assessed.

## ALCOHOLIC LIVER DISEASE AND HCC

**Figure 1.**
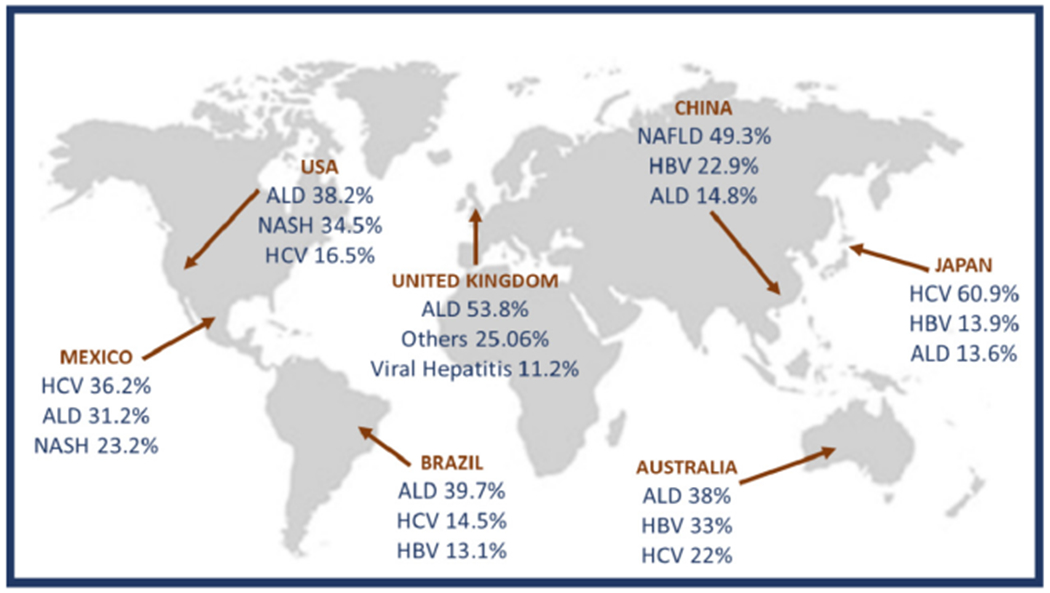
Worldwide prevalence of cirrhosis secondary to alcohol abuse compared with other cirrhosis etiologies. Modified from Méndez-Sánchez *et al*.^[9]^. HCV: hepatitis C virus; ALD: alcoholic liver disease; NASH: non-alcoholic steatohepatitis; HBV: hepatitis B virus; NAFLD: non-alcoholic fatty liver disease

For the development of ALD, the fulfillment of two factors is generally necessary; one is an excessive alcohol consumption, defined as ingestion of > 20 g/day in females and > 30 g/day in males, and the second one is the chronicity of this consumption^[3]^. On its own, ALD is one of the less frequent etiologies that progress to HCC^[6,7]^ however, its high prevalence continues to position it as one of the most important chronic liver diseases (CLDs) for public health^[8]^. Recently, our group of work conducted a study to determine the main etiologies of cirrhosis worldwide [Figure 1] finding interesting results among countries^[9]^.

In a healthy person, alcohol is metabolized to acetaldehyde mainly in the liver by the alcohol dehydrogenase (ADH) and the microsomal ethanol-oxidizing system (MEOS), and to a lesser extent it is also metabolized in the GI tract through ADH, MEOS, and the gut microbiota^[10]^. Several factors predispose the development and progression of ALD to its final stage of HCC, the most important being genetic predisposition, age, female sex, pre-existing liver disease, and daily alcohol consumption^[5]^. Similarly, the GI tract has its own factors that predispose the metabolism and systemic absorption of ethanol and therefore the severity of ALD. An example of this is the diminished enzymatic activity of ADH in the stomach commonly seen in young women, elderly, alcoholics, when fasting, and after treatment with H2-receptor antagonists. Other situations that favor systemic absorption of ethanol are delayed gastric emptying, chronic atrophic gastritis, and gastric lesion associated with *Helicobacter pylori*^[10]^. Nonetheless, in ALD, there is an increase in the metabolization of ethanol to acetaldehyde by the cytosolic enzyme ADH and then from acetaldehyde to acetate by the mitochondrial enzyme aldehyde dehydrogenase^[11]^. In the long run, this will generate mitochondrial dysfunction, which is considered a critical step for the onset and progression of ALD^[12]^. Dysfunctional mitochondrial can undergo a fragmentation pathway to further be cleared by autophagy or promote the apoptotic cascade in sever liver injury by a multi-step process called “mitochondrial dynamics” controlled by the activity of the mitochondria shaping proteins (MSP)^[13]^. In a recent study, Palma *et al.*^[14]^ demonstrated that mitochondrial dynamics showed important changes in alcoholic steatohepatitis (ASH) patients by finding an increased expression of the MSP protein dynamin-related protein 1 (DRP1) compared with controls. They also found a direct correlation between DRP1 mRNA levels and blood concentration of aspartate aminotransferase in those patients. Interestingly, this was only seen in advanced ALD subjects, suggesting the study of mitochondrial deregulation in ALD progression is an important issue.

On the other hand, high alcohol consumption has been related with increased MEOS activity and its first constituent, the cytochrome P-450 2E1 (CYP2E1)^[15,16]^. This has a great impact since, unlike the usual dehydrogenation process, the oxidation of ethanol by MEOS is carried out through several reactive intermediates known as reactive oxygen species (ROS) via CYP2E1^[17]^. An increase in alcohol consumption upregulates the activity of intestinal MEOS, leading to an increase in ROS production, which interferes with the barrier function of the gut^[17]^.

## MICROBIOTA AND ITS INTERACTION WITH THE INTESTINAL ENVIRONMENT

The GI tract is the natural habitat for several microorganisms, including bacteria, archaea, viruses, and parasites. In a healthy gut microenvironment, there is a predominant diversity of seven large groups: Firmicutes, Bacteroidetes, Actinobacteria, Fusobacteria, Proteobacteria, Verrucomicrobia, and Cyanobacteria^[18]^. The gut microbiome, which refers to the collective genomes of all the microorganisms that compose the gut microbiota, contains 150 times more genes than the human genome^[18]^ . In addition, gut bacteria has been appreciated for the benefits they can provide to the host (symbiosis) as they supply essential nutrients such as vitamins, metabolize non-digestible compounds, and even defend against pathogenic microorganisms^[19,20]^.

The colonization of the healthy gut environment contributes to the development of the intestinal architecture and the proper functioning of the immune system. Colon bacteria can ferment nutrients and endogenous substrates derived from the host, such as mucus and pancreatic enzymes, as well as dietary components that are not absorbed in the first portions of the GI tract. Thus, the gut microbiota produce and transform a wide variety of metabolites that are absorbed in the small intestine, which can then travel through the bloodstream and reach the systemic circulation, especially the brain and liver, where they can trigger or influence important signaling pathways^[21]^.

The gut is a large territory occupied by both commensal and pathogenic microorganisms; therefore, it has the important protective mechanism of selectively choosing which molecules may pass to the systemic bloodstream. This mechanism is established by a multi-layer intestinal barrier covered by a mucus layer that provides a physical barrier between the underlying epithelium and the GI tract. This intestinal barrier consists in two separate sub-layers: an inner layer attached to epithelial cells lacking bacteria and an outer layer colonized by commensal microorganism. In addition to protecting against harmful agents, it acts as a selective filter for the correct translocation of nutrients, electrolytes, and water from the intestinal lumen to the circulation^[22,23]^.

## Cell composition of the intestinal barrier

**Figure 2.**
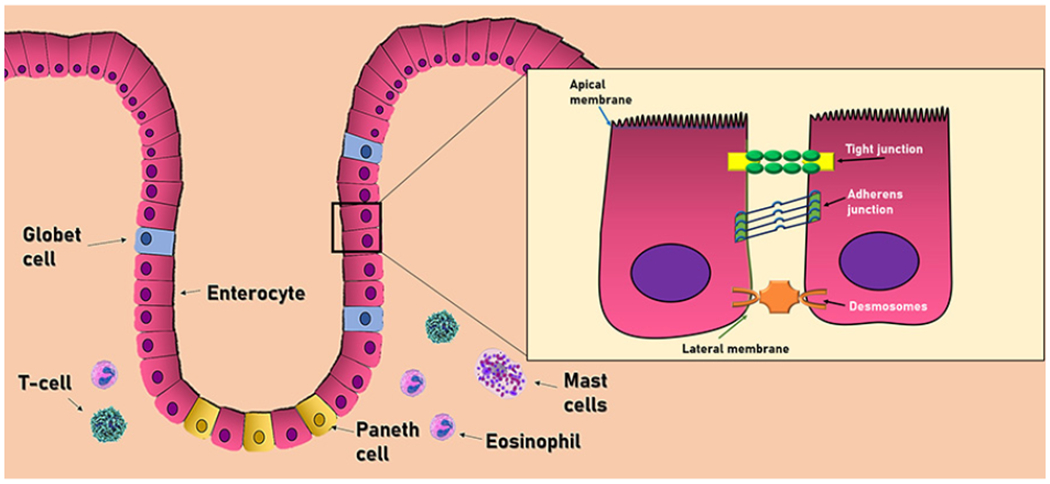
Cell composition of the intestinal barrier. The intestinal epithelium consists of a single layer of epithelial cells. Adjacent cells are connected by three main junctional complexes: desmosomes, adherens junctions, and tight junctions. The main immune cells of the intestinal barrier consist of T-cells, mast cells, and eosinophils

The intestinal barrier has three main cell types aimed to protect the host against external aggressions. This group includes the epithelial cells, intestinal goblet cells, and Paneth cells^[24]^ Epithelial cells form a physical barrier connected by many transmembrane proteins called tight junctions (TJ), adhesion junctions (AJ), and desmosomes, each located in the basolateral membrane of epithelial cells. The TJ (also called zonula adherens) are located in the most apical part, formed by the cadherin-catenin protein junction. Below this zone, in almost the entire extension of the basolateral membrane, we can find the AJ (also known as zonula occludens), formed by the union of three main proteins: occludins, claudins, and the junctional adhesion molecules (JAM). Occludin and claudins are responsible for biochemical permeability and cell adhesion, while JAM bind cells by anchoring to the actin cytoskeleton of each cell. Finally, desmosomes can be found in the lower area of the epithelial cells, which also provide junction points by using keratin filaments^[23]^ [Figure 2].

Intestinal goblet cells produce different types of mucins (Muc2, Muc5AC, and Muc6), contributing to the viscous properties of the intestinal mucus layer and the protection against the pathogens that penetrate this layer. Similarly, Paneth cells secrete the derived regenerating islet (Reg) 3β in the mucus layer. These molecules are involved in gut homeostasis and exhibit antimicrobial activity that shapes the composition of the intestinal microbiome^[22]^. All these gut defense mechanisms are reinforced by numerous immune cells in the *lamina propria* that play an essential role in the protection of the intestinal mucosa against the invasion of bacteria. Of this large number of immune cells, it is worth highlighting T cells, mast cells, and eosinophils due to their important contributions^[23]^. First, T cells regulate cell permeability through Na^+^/K^+^ ATPase pumps, as well as the release of proinflammatory cytokines such as interferon-gamma (IFNγ), tumor necrosis factor-alpha (TNF-α), and delta-positive intestinal intraepithelial lymphocytes (iIELγδ+), which are also found in the basolateral membrane of epithelial cells, involved in the maintenance of its function. Mast cells release different proinflammatory mediators such as histamine, leukotrienes, plateletactivating factor, and cytokines, with important immune-mediated functions throughout the entire GI tract. Ultimately, eosinophils increase intestinal permeability through different mediators such as histamine, prostaglandins, and TNF-α^[22]^.

## Composition of a “healthy” gut microbiota

**Figure 3.**
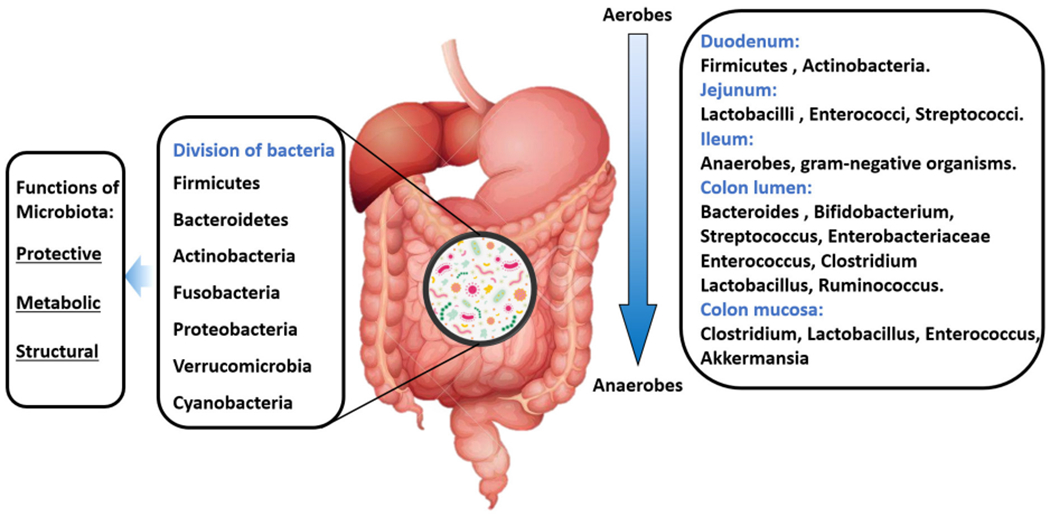
Composition of a “healthy” gut microbiota

In the small intestine, food and nutrients absorption is mainly done in the duodenum through the release of digestive enzymes. At this site, food transit is faster, and the presence of oxygen limits bacterial density [10^[3–4]^ Colony-forming unit (CFU)/mL], Firmicutes and Actinobacteria predominate in this site with an important growth of Gram-positive aerobes and facultative anaerobes, including Lactobacilli, Enterococci, and Streptococci with a progressive increase in bacterial density (10^[3–7]^ CFU/mL) in the jejunum^[25]^. In the first part of the ileum, the bacterial density increases with a predominance of aerobic species (10^[9]^ CFU/mL). In contrast, the distal part of the ileum (near the ileocecal valve) is inhabited by anaerobes and Gram-negative microorganisms similar to those found in the colon (characterized by a slower transit and its anaerobic condition). In the colon, the number of anaerobes exceeds aerobes microorganisms with a bacterial density of 10^[12]^ CFU/mL and an important predominance of Firmicutes and Bacteroidetes. Moreover, in the GI lumen, *Bacteroides, Bifidobacterium, Streptococcus, Enterobacteriaceae, Enterococcus, Clostridium, Lactobacillus* and *Ruminococcus spp*. are the bacterial genera that predominate, while *Clostridium, Lactobacillus, Enterococcus*, and *Akkermansia spp*. are more frequent in the mucosa [Figure 3]. In addition, some pathogenic bacteria including *Campylobacter jejuni, Salmonella enterica, Vibrio cholera, Escherichia coli*, and *Bacteroides fragilis* can be found in smaller amounts within the GI tract^[25]^.

## OXIDATIVE STRESS AND INTESTINAL PERMEABILITY IN ALD

When there is an increase in alcohol consumption, an upregulation of the CYP2E1-dependent ROS products such as hydroxyethyl, superoxide anion, hydroxyl radicals and numerous free radicals will accumulate in the liver, developing oxidative stress. An accumulation of ROS produces structural and functional changes in the DNA that interfere with the cell cycle, playing an important role in carcinogenesis^[11]^. One of these changes induced by acetaldehyde and ROS is related to epigenetic regulations by interfering with the folate metabolism (important for DNA synthesis and methylation)^[26]^. ALD patients have been found with polymorphisms in the methylene tetrahydrofolate reductase gene, leading to an alteration in folate metabolism and HCC development^[27,28]^. Alcohol also has the capacity to inhibit the synthesis of S-adenosyl-L-methionine (SAMe), an important methyl-donor molecule, by a diminished activity of methionine adenosyltransferase. The consequence of chronic SAMe depletion seems to be associated with liver injury by interfering with the regenerative capacity of the liver^[29]^. Furthermore, oxidative stress induces lipid peroxidation products such as malondialdehyde and 4-hydroxy-2-nonenal with the capacity to modify the gut microbiome, enhancing the creation of endotoxins by gut bacteria^[30]^, as well as induce mutations in the p53 gene, promoting HCC development^[31]^.

In the same way, evidence suggests that intestinal MEOS plays a permissive role in the gut, probably by the integrity disruption of the narrow epithelial junctions, which induces a decreased expression of binding proteins (mainly claudins) with the consequent dysfunction of the AJ, establishing a leaky gut^[32]^. Rodent studies have also demonstrated that alcohol-associated intestinal permeability is favored by a reduction in the intestinal hypoxia-induced factor 1-alpha (HIF-1α) activity, a condition reversed by probiotic *Lactobacillus rhamnosus GG* supplementation^[33,34]^. Moreover, ALD patients show a decreased bacterial diversity associated with an increase of endotoxin-producing Enterobacteriaceae and Proteobacteriaceae and a reduction in taxa that produce short-chain fatty acids such as Lachnospiraceae, Bacteroidaceae, and Ruminococcaceae^[35–37]^. Interestingly, a reduced expression of lectins Reg3fl and regenerating islet-derived protein 3 gamma (Reg3γ) is another important characteristic commonly seen in ALD, associated with bacterial overgrowth and translocation^[38]^. All these factors will induce endotoxins formation such as lipopolysaccharides (LPS), peptidoglycans, and bacterial DNA. This favors intestinal inflammation and the activation of the TNF-α receptor I signaling in intestinal epithelial cells associated with increased intestinal permeability of endotoxins to the liver, boosting systemic inflammation via recognition of specific toll like receptors (TLRs)^[39,40]^, as discussed below in more detail. Moreover, commensal fungi such as *Candida spp*., *Saccharomyces cerevisiae*, and *Malassezia spp*. will develop tolerance from the host immune system during chronic alcohol consumption, fomenting an increase in these fungal species^32]^. Interestingly, studies in ALD patients have also shown higher systemic endotoxemia levels in subjects with an increased alcohol consumption regardless of the stage of liver disease, demonstrating that alcohol consumption is an independent factor for systemic endotoxemia^[41,42]^.

## MECHANISMS INVOLVED IN HCC DEVELOPMENT

In the liver, Kupffer cells and bone-marrow derived macrophages will recognize small sequences of molecules formally called pathogen-associated molecular patterns (PAMPs) from endotoxins coming from enterohepatic circulation via Toll-like receptor-4 (TLR4). The upregulation of TLR4 will promote binding with its ligand, myeloid differentiation primary response 88, resulting in the activation of c-Jun N-terminal kinase, the inhibitor of nuclear factor kappa-B kinase 2, and mitogen-activated protein kinase (MAPK) p38, with the consequent activation of the nuclear factor kappa-B (NF-κβ) pathway. This favors the release of TNF-α, IFN-γ, prostaglandin-2, chemokine C-C motif ligand, IL-1α, IL-1β, IL-6, ROS, and nitric oxide, perpetuating liver inflammation^[43]^. NF-κβ can also induce the antiapoptotic genes (TRAF-1 and TRAF-2) with important carcinogenic effects^[4]^. Increased TNF-α production has been shown to deregulate TJ, causing disruption of the intestinal barrier. Interestingly, high levels of TNF-α and IL-6 have been found in duodenal biopsies of alcohol-dependent subjects, which tend to confirm data obtained in animal models^[28]^. In another study carried out in 52 subjects diagnosed with alcohol dependence according to the DSM-IV criteria, a biochemical panel measuring LPS, TNFα, IL-6, IL-10, and high C reactive protein sensitivity showed an important elevation of these biochemical markers^[44]^. On the other hand, IL-37 has been associated with anti-inflammatory effects via IL-18Rα and IL-1R8 expression. In liver samples of ASH subjects, IL-37 expression was substantially reduced when compared to non-alcoholic fatty liver disease subjects^[45]^. An *in vivo* system in wild-type mice suggested that hepatic IL-37 expression was suppressed by ethanol through the administration of human recombinant IL-37 followed by oral gavage of an ethanol shot in those animals^[45]^. This is important since HCC clinical specimens have shown that decreased expression of IL-37 is negatively correlated with tumor size and positively associated with better overall survival and disease-free survival via the induction of tumor-infiltrating CD571 natural killer cells^[46]^.

In the liver, TLR4 can also be expressed in hepatic stellate cells (HSCs), endothelial cells, and hepatocytes^[47]^. In HSCs, this molecule is involved in the upregulation of hepatocytogen epiregulin^[48]^, an epidermoid growth factor with a potent mitogenic effect on hepatocytes^[49]^. In conjunction with the antiapoptotic effect of NF-κβ, it significantly promotes the hepatocarcinogenesis process. Knock-out mice studies with TLR-4 deficiency and intestinal sterilization with non-absorbable antibiotics have found a reduction in steatosis, oxidative stress, and liver inflammation with a consequent decrease in HCC risk development^[50,51]^, although the risk for liver injury increased, probably due to a deficiency in the innate immunity caused by the suppression of TLR-4. In addition, chronic alcohol consumption has been associated with immunosuppression though a reduced recruitment of CD8^+^ T cells, an important group of cells responsible for the anti-tumor response in the human body^[52]^.

## CHANGES IN THE GUT MICROBIOTA OF HCC PATIENTS

The gut microbiota undergoes an important change in the guests with early HCC. In obesity-induced mouse models, a greater number of Clostridium species has been found^[53,54]^, while in humans an important growth of Escherichia coli^[55]^, Actinobacteria, Gemmiger, and Parabacteroides species^[56]^ has been reported. In addition, due to the large number of bacteria that coexists in the body and the bacterial translocation caused by a leaky gut, it is not uncommon to find metabolically active bacteria within richly vascularized tumors attracted through a chemotactic gradient of the necrotic cell debris^[57]^. In the case of HCC, Helicobacter species have been found with some frequency in this type of tumor tissue^[58–60]^. In fact, this relationship is so important that an influence of the gut microbiota in the effectiveness and toxicity of certain chemotherapeutic agents has been pointed out, especially with the immune checkpoint inhibitors through the interaction among PAMPs, antigen-presenting cells, and TLRs, which leads to an adaptive immune response that modifies the pharmacodynamics of these types of agents^[57]^. Moreover, both animal and human studies have found a significant correlation between alcohol consumption and a disturbance in the Lactobacillus to Bifidobacterium ratio, with an increase in pathogenic bacteria (namely, Proteobacteria and Bacilli). Interestingly, this ratio derangement has different presentations according to alcohol consumption habits, duration, and liver disease stage^[61,62]^

Looking at other examples of HCC development related to microbiota imbalances in hepatology, we can describe the evidence regarding chronic viral hepatitis B and C. Chronic hepatitis B (CHB) patients show lower bacterial diversity (namely, an increase of Firmicutes and a decrease of Bacterioidetes). There is an increased concentration of H_2_S- and CH_3_SH- producing phylotypes (Fusobacterium, Filifactor, Eubacterium, Parvimonas, and Treponema) that may produce small bowel bacterial overgrowth, potentially involved in cirrhosis and HCC development^[63]^. However, the impact of gut microbiota derangements in CHB patients on hepatocytes neoplastic transformation is different from that of chronic hepatitis C patients^[64]^. In fact, obesity and/or diabetes stimulate cellular oncogenesis via gut microbiota derangement (i.e., an abundance of Bacteroidetes and, at a genus level, Prevotella, Acinetobacter, Veillonella, Phascolarctobacterium, and Faecalibacterium abundance) in HCC patients^[65–67]^.

Moreover, both interferon and new interferon-free direct antivirals successfully treated HCC patients presenting a permanent chronic inflammatory state triggered by an altered gut microbiota with potential HCC promotion^[68–70]^.

## MOLECULAR INVOLVEMENT OF THE BILE ACIDS

Bile acids (BAs) are amphipathic molecules obtained from cholesterol synthesized in the liver, which play an important role in the emulsification of fats obtained from the diet to facilitate their absorption, in addition to important regulatory effects on the signaling pathways of glucose, lipids, and amino acids^[71]^. In a healthy host, most of the BAs’ pool is reabsorbed by active transport in the terminal ileum, while the rest is dehydroxylated by the intestinal microbiota, such as the secondary BAs deoxycholic acid (DCA) and lithocholic acid^[72]^.

The disruption in bacterial diversity of the host induced by ALD brings with it an important change in the BAs’ pool by upregulating bacterial dehydroxylation, resulting in an increase in DCA synthesis, known for its important cytotoxic and carcinogenic effects. It is known that, under conditions of accumulation of BAs, activation of farnesoid X receptor (FXR) induces the expression of the bile salt export pump, organic solute transporter alpha, and organic solute transporter beta, promoting the efflux of hepatic and intestinal BAs to systemic circulation^[73]^. However, in CLDs, a decrease in these transporters has been observed due to an inhibition in FXR signaling by the subunit NF-κβ p65 binding directly to FXR, which inhibits its transcriptional activity, thus maintaining liver inflammation and the probable development of HCC^[74]^. In addition, DCA can disrupt the plasma membrane, causing activation of protein kinase C, which in turn activates p38 MAPK, increasing the activation of NF-κβ pathway and resulting in sustained inflammation^[75]^. Furthermore, the NF-κβ pathway transcribes genes encoding pro-inflammatory cytokines such as IL-6 related to the activation of the signal transducer and activator of transcription 3 pathway, which leads to decreased apoptosis^[76]^, and IL-1β related to the activation of phosphoinositide 3 Kinase-MDM2 pathway, which negatively regulates p53, thus increasing the survival of DNA-damaged cells and leading to the development of HCC^[77]^.

Finally, recent findings have suggested an important role of DCA and cellular senescence in the development of HCC^[53]^. Cellular senescence is a protective cell response to telomere erosion or oncogene activation with the final objective of bringing to an end the compromised cell cycle to prevent the development of any neoplasm^[78]^. Interestingly, senescent cells develop a secretory proinflammatory profile known as senescence-associated secretory phenotype (SASP)^[79]^. An experimental model in mice found that DCA induces SASP phenotype in HSCs, which in turns favors the secretion of proinflammatory cytokines and tumor-promoting factors associated with HCC development^[53]^. It should be noted that this was an obesity-induced mice model; nonetheless, the results of this study could be replicated in an animal model of high-alcohol consumption to determine if there is any important variation between models^[53]^.

## MICROBIOTA-REGULATORS AS A THERAPEUTIC OPTION FOR HCC

Due to the close relationship between dysbiosis and HCC, it is not difficult to imagine that certain microbiota-regulating agents have been used in several experimental studies in both humans and animals showing encouraging results. In this context, the drugs that have shown greater efficacy are the non-absorbable antibiotics rifaximin^[80–84]^ and norfloxacin^[85–87]^ by presenting an increase in the survival of patients with cirrhosis and HCC, in addition to preventing associated complications such as hepatic encephalopathy, portal hypertension, and spontaneous bacterial peritonitis. Other drugs included in this therapeutic arsenal are probiotics due to their modulating effects on the gut microbiota, by trying to restore bacterial diversity^[88]^. Unfortunately, many pharmaceutical and food companies have made significant profits with them, which is why many so-called “healthy bacterial compounds” can be found in both pharmacies and supermarkets, making it difficult for health authorities to regulate them. Another important option that has not proven its efficacy in cancer but has in other GI conditions such as *Clostridium difficile* infection is fecal microbiota transplantation, promising to “reset” the altered microbiota, thus improving the anti-cancer immune response and preventing its development^[89]^. Unfortunately, all these therapeutic options are still not included in the guidelines for the management of HCC due to the lack of standardization in different populations; thus, new clinical studies that focus on the resolution of intestinal dysbiosis for the management of HCC are necessary to increase its therapeutic options.

## CONCLUSION

ALD is one of the most prevalent CLDs worldwide, representing a major health problem for most countries. Although it has a low potential for malignancy compared to other CLDs, its wide prevalence represents a major health problem for most countries. In recent years, great advances have been made in this field. To date, we know that alcohol metabolites interfere with the mitochondrial regulation pathways via increased expression of MSP, representing an attractive research field for understanding ALD pathogenesis. In addition, alcohol has the capacity to disturb gut microbiota, favoring the expansion of endotoxin-producing bacteria and intestinal permeability, with the final translocation of bacteria and bacteria metabolites to the liver, inducing liver injury and carcinogenesis via the recognition of TLR-4 and the activation of NF-κβ pathway. Microbiota-regulating drugs have proven an important efficacy in the survival of patients with cirrhosis and HCC. However, alcohol abstinence will always be the best option for these patients; thus, emphasis should be placed on dissemination programs that teach the population about the important complications derived from alcohol consumption.

## Figures and Tables

**Figure 4.**
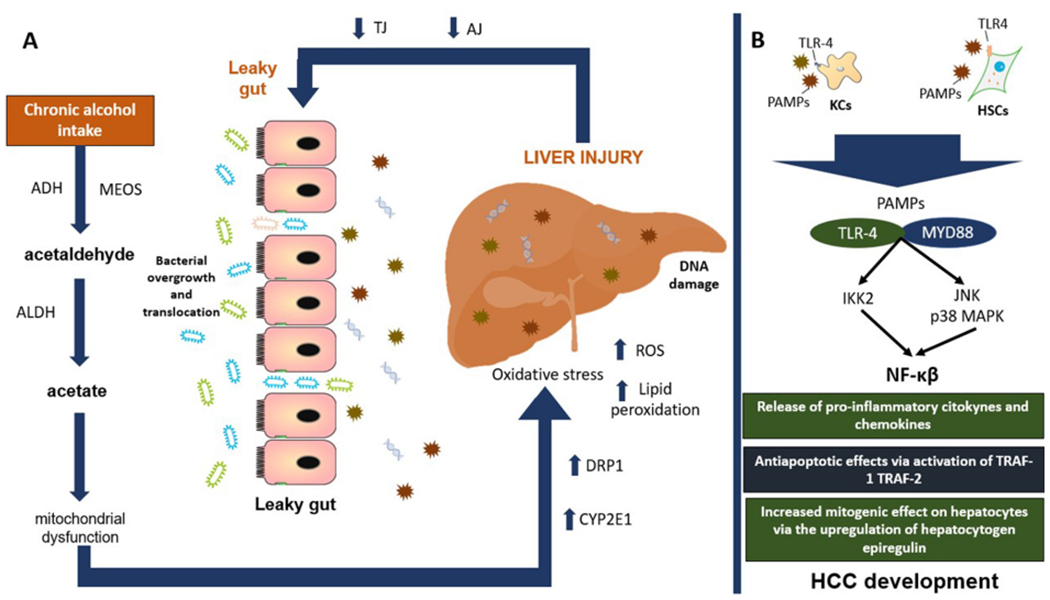
Main mechanisms involved in the development of HCC. A: chronic alcohol consumption increases the production of its main toxic metabolite acetaldehyde, favoring mitochondrial dysfunction and oxidative stress perpetuating liver injury. In the long run, this will generate a decreased function of TJ and AJ, interfering with the protective barrier of the intestine, developing a leaky gut. B: we can see how the bacterial overgrowth and translocation of its metabolites to the liver will increase liver injury and the recognition of PAMPs by specific TLRs such as TLR-4 binding with its ligand MYD88 and with the final activation of NF-κβ pathway with important repercussion for systemic inflammation and HCC development. ADH: alcohol dehydrogenase; MEOS: microsomal ethanol oxidizing system; ALDH: aldehyde dehydrogenase; DRP1: dynamin-related protein 1; CYP2E1: cytochrome P450 2E1; ROS: reactive oxygen species; TJ: tight junction proteins; AJ: adhesion junction proteins; PAMPs: pathogen-associated molecular patterns; TLR4: toll-like receptor-4; KCs: kupffer cells; HSCs: hepatic stellate cells; MYD88: myeloid differentiation primary response 88; JNK: c-Jun N-terminal kinase; MAPK: mitogen-activated protein kinase; IKK2: inhibitor of nuclear factor kappa-B kinase 2; NF-κβ: nuclear-factor κβ; TNF: tumor necrosis factor; TRAF-1: TNF receptor associated factor-1; TRAF-2: TNF receptor associated factor-2; HCC: hepatocellular carcinoma
